# Feasibility of sustainable provision of intradermal post exposure prophylaxis against rabies at primary care level –evidence from rural Haryana

**DOI:** 10.1186/1472-6963-14-278

**Published:** 2014-06-25

**Authors:** Harshal Salve, Sanjeev Kumar, Rizwan SA, Sanjay K Rai, Shashi Kant, Chandrakant S Pandav

**Affiliations:** 1 Centre for Community Medicine, Old OT Block, All India Institute of Medical Sciences, Ansari Nagar, New Delhi 110029, India; 2 Department of Community Medicine and Family Medicine, All India Institute of Medical Sciences, Bhopal, India

**Keywords:** Intra-dermal PEP, Rabies, Primary care, Rural, India

## Abstract

**Background:**

Rabies is the most severe and neglected public health problem in India. Management of animal bite with post exposure prophylaxis is the only existent strategy to prevent rabies related deaths. Cost-effective and sustainable programme for provision of post exposure prophylaxis (PEP) is needed in India.

**Methods:**

In this study, we have documented the experience of implementation of intra-dermal anti rabies vaccination in Animal Bite Management (ABM) clinic at Primary Health Centre (PHC). This study facility belonged to Comprehensive Rural Health Services Project, Ballabgarh in Faridabad district of Haryana. Hospital service record of ABM clinic was analyzed and various feasibility issues such as costing of services, vaccine wastage and other operational issues in providing PEP services at PHC level were documented.

**Results:**

A total of 619 patients were treated in the ABM clinic. Service utilization of ABM clinic was increased by 38% in the second year of implementation. Mean age of the patients was 23.9 years (SD: 18.8) and majority (70.4%) were males. Majority (86%) of the patients received the first dose of anti-rabies vaccine within the recommended 48 hours. A total 446 vaccine vials (1 ml) were consumed of which 20.8% was contributed in vaccine wastage. User-fee (350 Indian Rupees) collected from the patients. User-fee was re-used to purchase vaccines, intradermal (ID) syringes and other consumables required to ensure regular availability of ARV services at the PHC.

**Conclusions:**

This study demonstrated the cost-effective and sustainable model of provision of PEP against rabies at primary care level. ID PEP provision at primary care level not only address the unmet need of animal bite management in the community also reduces the out of pocket expenditure of the patients.

## Background

Globally, annual incidence of human rabies is estimated to be between 30,000- 70,000 with more than 90% of cases reported from developing countries. This contributes to 20,000 deaths attributed to Rabies and 17.4 million cases of animal bite per annum. The highest numbers of human deaths due to rabies are observed in India and Philippines [1]. India accounts for 36% of the Global and 65% of the Asian rabies related deaths [2]. In India, a nationwide survey reported that annual incidence of animal bites was 1.7% (2003), more common in rural areas, among children and low income groups [2]. In spite of this disease burden, rabies is a neglected infectious disease in India. The main biting animals are stray dog followed by cat [2]. Rabies can be averted only by effective pre- exposure prophylaxis (PrEP) or post-exposure prophylaxis (PEP) [3]. PEP includes anti-rabies vaccine administration, and for severe categories of exposure, infiltration of purified rabies immunoglobulin (RIG) in and around the wound [4]. In low and middle income countries, RIG is rarely used as it is expensive [5,6] and not regularly available [7,8]. Hence, only post exposure vaccination is provided to patients with animal bites [9]. In the year 2007, out of all animal bite cases, 50% received PEP in India. Out of this almost 40% got nerve tissue vaccine (NTV) due to its low cost and free availability [8]. Government of India (GOI) banned use of NTV in the year 2004, since then patients of animal bite are forced to purchase Tissue Culture Vaccine (TCV). Non-availability or irregular supply in public health system and high cost of TCV contributes to significant out-of-pocket expenditure (OOP) in the range of 1500-1800 Indian Rupees (Rs) for five doses under PEP. High OOP limits health care seeking by most of the animal bite-patients. In 1997, World Health Organization (WHO) recommended intra-dermal (ID) TCV administration in resource-poor setting [10]. Immunological response and effectiveness of PEP via intra-dermal route has been found to be similar to other intra-muscular regimens [11]. In the year 2006, GOI recommended use of ID TCV administration under PEP [12]. Although some Indian states provide TCV at free of cost through few public health facilities, erratic supply reduces its utilization. Loss of wages due to required multiple visits to the health facility has been incriminated in poor compliance to PEP. This has been also identified as a reason for increased rabies deaths especially in rural areas [12]. Therefore, the possibility of PEP provision for rabies via self-sustaining mechanism at primary care level becomes worth exploring.

As an initiative under National Rural Health Mission (NRHM), a patient welfare society or Rogi/Swasthya Kalyan Samiti (RKS/SKS) has been formed at all Primary Health Centres (PHCs) in India. The SKS is a registered society with the Medical Officer (MO) as the Chairman and selected healthcare provider and civil society representatives as members. Some funds are given to these SKSs by GOI through NRHM [13]. SKS members are authorized to utilize the funds. These societies can also levy user-fees which can be used for welfare of patients seeking healthcare. Encouraged by this financial flexibility, we planned to offer a comprehensive care package for patients with animal bite. The objective of this study is to document various feasibility issues related with provision of rabies ID-PEP services at a rural Primary Health Centre (PHC) in Haryana state in north India.

## Methods

Present study was carried out in one of the PHCs under Comprehensive Rural Health Services Project (CRHSP) Ballabgarh of All India Institute of Medical Sciences, New Delhi [14]. Since more than three decades, preventive, promotive and curative services had been provided through this PHC (Chhainsa) to the population of 47, 000 (year 2012). Curative services were provided through daily general outpatient clinic at the PHC. In the latter half of the year 2010, we realized that many patients were approaching the PHC seeking treatment for animal bites. At that time, they were provided first aid and referred to district hospital for PEP. The district hospital was 30 kilometers from the PHC. The distance and OOP expenditure led to many of the patients not seeking further care as reported by health workers, Accredited Social Health Activists (ASHA), and the family members themselves. At that time both government hospitals and private practitioners were following intramuscular regimen (days 0-3-7-14-28 – 90) for PEP without RIG for animal bite management.

An Animal Bite Management (ABM) clinic was started at the PHC in December 2010. Purified Chick Embryo Cell (PCEC) Vaccine and ID syringes were purchased using the funds provided by NRHM to SKS. We followed a two site (both deltoid regions), four doses (0, 3, 7, 28 days), intra-dermal regimen known as the ‘*Up-dated Thai Regimen’*[11]. In this regimen, 0.1 ml ARV was given at both arm at deltoid region. Each patient received 0.2 ml ARV on vaccination day. We considered Day 0 as the day of administration of first dose of ID rabies vaccine, and this may or may not be the day of animal bite. Day 14 was skipped as compared to the IM regimen. In case of a bite-site on the arm, the vaccine was given by the ID route on supra-scapular areas [5]. Since the patient load was expected to be small at the beginning of this service, we decided to offer vaccination on fixed days (Mondays, Wednesdays and Saturdays). The schedule was made so. If a patient reported on other days, and if the next vaccination day was within 2 days (48 hours) of the date of exposure, patient was called on the that day for PEP.

In the ABM clinic, we followed WHO protocol for animal bite management. After confirmation of diagnosis, we provided thorough wound washing with soap and running water, wound care and *tetanus* prophylaxis (if not given before). We collected information on socio-demographic characteristics; earlier history of animal bite and PEP, selected characteristics of the biting animal. The provider then assessed the wound and decided on PEP schedule including other symptomatic treatment and antibiotics. Advices were given emphasizing the severity of rabies and the need to adhere to the schedule, do’s and don’ts in case of animal bite. For cases requiring RIG, referral was made to the district hospital. The SKS committee decided to levy Rs. 350 from users of ABM Clinic services. Very poor patients, widows and patients belonging to Below Poverty Line (BPL) were exempted from the payment of user-fee.

### Record keeping

Record of the patients in the form of brief exposure history, previous PEP history and socio-demographic information was maintained. This record was obtained and maintained by the staff nurses and ANM posted at the PHC. Contact numbers of the individual patients were collected to do follow up and to remind patients of PEP scheduled date in case of default. In the follow up, information about any vaccine related side effects and rabies related deaths were obtained on telephone. Record of user-fee collected was maintained by the medical officer in-charge. A separate account in a nationalized bank was maintained by the medical officer in-charge on behalf of SKS to deposit the amount so collected from PEP. The SKS commissioned yearly audit of account by the authority from district headquarter according to NRHM guidelines.

### Training of PHC staff

ID PEP was administered by staff nurses/ANM posted at the PHC. These staff nurses/ANM were involved in routine vaccination programme and had adequate technical skills in ID administration of ARV which was reinforced from time to time. Sensitization of PHC staff regarding cause of rabies, transmission of rabies, preventive measures and ID PEP was carried out by the medical officers posted at the PHC [5].

### Information Education and Communication (IEC)

**Figure 1:**
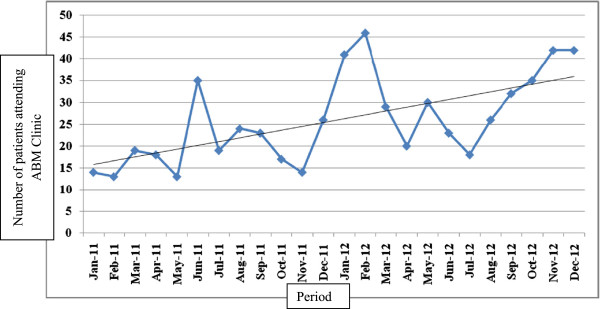
Distribution of ABM clinic attendee by period (Jan 2011 to Dec 2012).

Health workers, ASHA, Anganwadi worker (AWW) and opinion leaders of the community such as village headman (Sarpanch), school teacher etc. were involved in IEC. This helped in improved use of ABM services at the PHC (Figure 1).

In this paper, we have described our experience of ABM clinic from January 2011 to December 2012. We explored various feasibility issues such as cost effectiveness, vaccine wastage and other operational issues in providing PEP services at PHC level.

### Ethical issues

Ethical clearance for the study was obtained from Ethical Committee of All India Institute of Medical Sciences, New Delhi.

## Results

### Socio-demographic profile of patients attending ABM clinic

A total of 619 patients were treated in the ABM clinic of PHC. Service use of ABM clinic was increased by 38% in the year 2012 as compared to year 2011. Patient burden at ABM clinic showed increasing trend during study period (Figure 1). Mean age of the patients was 23.9 years (SD: 18.8) and majority (70.4%) were males (Table 1).

**Table 1:** Socio-demographic profile of the patients attended ABM clinic (N = 619)

**Age group (years)**	**Number**	**Percentage**
<=18 years	303	48.9
19 to 35 years	179	29.0
36 to 59 years	94	15.2
> = 60 years	43	6.9
**Sex**		
Male	436	70.4
Female	183	29.6
**Distance of residence from study facility (PHC)**	**Number**	**Percentage**
< 5 kms	245	39.6
> 5 kms	374	60.4

### Characteristics of the treatment availed at ABM clinic

Nearly 495 (80%) of the wounds were bleeding wounds (Class III). In about 99 (16%), the skin was just broken (Class II), and in 25 (4%) the skin was intact (Class I). Majority 533 (86%) of the patients received the first dose of ARV within the recommended 48 hours. Mean (SD) time interval between exposure and first dose of ARV was 2.2 days (SD: 4.2). Of total, 452 (73.0%) patients completed full course of PEP. Among the patients who completed treatment, 375 (82.8%) adhered to the schedule. None of the patients received PEP reported with any severe complication due to vaccine or rabies related death during one year post vaccination.

### Consumption of vaccines and other consumables

During study period, 619 animal bite-patients reported to the ABM clinic, and a total 446 vaccine vials (1 ml) were consumed for PEP. One vial was used to vaccinate five patients (0.2 ml each) on the scheduled vaccination day. As per the recommendations of the manufacturer opened vial was used within 8 hours of reconstitution. The remaining vaccine, if any, was thereafter discarded. This resulted in 892 ID doses (~90 vials) being wasted. In total, 1890 ID syringes were used during study period for PEP.

### Vaccine storage

Vaccine was stored in Ice-lined refrigerator (ILR) at 4 to 8 *Degree Celsius* along with other vaccines under routine immunization programme*.* ILR with capacity of 70 litters was found to be enough for storage of 100 vaccine vials at any given point of time. The ANM posted at PHC maintained the temperature record for ILR twice daily. Record showed that temperature was maintained at 2 – 8 degree Celsius throughout the study period.

### Costing of the services

ARV was not available through government supply. In the initial period, vaccine was bought through the funds available at PHC under SKS funds following prescribed norms. The cost of one vial that contained 1 ml or 5 ID doses was Rs. 300. User-fee (Rs. 350) collected from the patients was re-used to purchase vaccines, ID syringes and other consumables required to make sure regular availability of PEP services at the PHC. One vial and four ID syringes were used to complete PEP schedule for one patient. Hence, total average cost incurred for provision of PEP to one patient was Rs. 315. Thus we gained Rs. 35 from each user charge paying patient. This mechanism helped us to offer this service to poor patients free of cost and to compensate for vaccine wastage. During study period, 76 (12.3%) patients were exempted from the user-fee. Vaccine wastage cost around Rs. 2700 during period of two years. Other services such as tetanus toxoid, first-aid and antibiotics were provided free of cost to all the patients.

### Burden on existing health staff providing the services

Daily patient (old + new) load at ABM clinic was 20-25. Nursing staff had to spend 6-8 minutes per patient for ARV administration and record filling. This resulted in 120-200 minutes per ARV vaccination day. Medical Officer In-charge had to spend approximately 24 hours per month for supervision and procurement of vaccines and other consumables for ABM clinic.

## Discussion

In this study, we have documented our experience of providing of Rabies PEP and related care at primary care level in rural community of north India. The PCEC vaccine used in the ABM clinic has been considered to generate equal immunological response as that of HDC vaccine but at a cheaper rate [15]. Intradermal *Up-dated Thai Regimen* (2 -2 -2-0-2) was used for PEP whose safety and immunogenicity is well documented in Indian population [16]. Usefulness and cost-effectiveness of ID ARV among population who cannot afford it is also well documented [17]. High attendance in ABM clinic reflected acceptance and utility of ARV services at primary care level. This programme can serve as a model for delivery of this service in resource-poor setting using the nationally approved funding and treatment guidelines. Drug Controller General of India (DCGI) has already approved PCECV for use of reduced dosage intra-dermal vaccination regimen in rabies PEP [14].

The NRHM has endorsed user-fee strategy to create local resources which would then be utilized locally to improve service availability and quality [13]. In developing country like India, user-fee mechanism affects the utilization of health service by increasing OOP expenditure and limitation to access to health service by ability to pay [18]. Patients had to pay 75% - 80% less amount to avail PEP from the PHC as compared to that of private practitioners who usually charged Rs. 1800 to Rs. 1900. If ARV services were available at district hospital free of cost, then patient from study area had to travel approximately 20-30 kilometers to avail the service. For travel by usually available mode of transport the patient would have spent approximately Rs. 40 - Rs. 60 for each of the five visits. Hence, an amount of Rs. 200 - Rs.300 travel would have been incurred by the patient on account of travel cost. Most of the patients of ABM clinic were daily wage laborer; hence availing services at district hospital would have cost loss of daily wage Rs. 200 - Rs. 300 per visit. Patient had to make five visits to complete the ARV IM schedule. Hence, total OOP for the patient due to animal bite could have been Rs. 1200 to Rs. 1800. However, this study demonstrated 70% - 80% reduction in OOP expenditure of patients on ARV. Also, indirect cost involved in terms of man-hour cost, travel time and expenses per visit was reduced. This was also evident from yearly increase in ABM clinic service use at PHC in this study.

Majority of patients that attended ABM clinic were of younger age group and males. The incidence of animal bites has been reported to be higher in these groups [3]. Majority of the attendees reported residing far from the PHC (>5 kms away). This could be attributed to widespread unmet need and acceptability of ARV services in the community. One fourth of the patients did not complete entire PEP regimen. Similar findings were reported by Rasania et al. in their study at Primary Health Center in New Delhi [19]. This could be due to the need of multiple visits to health facility and hence having economic consequences to the patients.

In this study, ID ARV was provided by nursing staff posted at PHC who were already trained in ID BCG vaccination in routine immunization programme. Hence, availability of healthcare provider for ID route of administration would not be an issue if the model is emulated elsewhere in the country in similar setting. The ABM services can be provided as part of the existing primary health setting and no extra human resources and funds need to be mobilized.

In the initial phases of clinic, patient load was low. ABM clinic was operational for three days week (Monday, Wednesday and Saturday) (Figure 1). One of the limitations encountered with this method was that the day 3 dose fell on the 2^nd^ day for patients receiving the first dose on Mondays and Saturdays. However this was essential to minimize vaccine wastage. Also, significant amount of duty time (8 – 10 minute per person) of staff nurse/ANM was expended for PEP on vaccination day. Anti-rabies passive prophylaxis was not provided due to high cost. But needy patients were referred to higher centers for the same. None of the previous study in India has reported use of anti-rabies antibody in public health system perhaps due to non-availability in government supply and high cost [2,20].

PEP service was not provided on all days in week to minimize the vaccine wastage. This limitation could be overcome by providing this service on all days a week provided patient load increased to an optimum level. Exemption from user-fees was provided on the basis of availability of BPL cards irrespective of actual socio-economic status of the family. This limitation was also documented in studies evaluating user-fees dependent services [20]. This financial mechanism will not be relevant where regular ARV is provided through government supply at primary care level as prevalent in many states of India.

## Conclusions

This study provides initial evidence of sustainable and cost effective model of PEP against rabies services provision at primary care level where its availability through government supply is a problem. It can also be argued that provision of such services may increase the trust of public in government healthcare system and increase the utilization of other services available but not popular at primary care level. Provision of services at primary care level will be one the way to attract population towards public health sectors.

## Competing interests

The authors declare that they have no competing interests.

## Authors’ contributions

HS, SK, SKR, SK conceived and designed the study, HS, RSA contributed in acquisition of data, data analysis and interpretation of data, HS & SK drafted the manuscript. SKR, SK & PCS provided critical inputs to the manuscript. All authors approved the final manuscript.

## Pre-publication history

The pre-publication history for this paper can be accessed here:

http://www.biomedcentral.com/1472-6963/14/278/prepub
